# Multifocal fatty liver nodules mimicking a metastatic disease: A case report

**DOI:** 10.1016/j.radcr.2023.11.040

**Published:** 2023-12-15

**Authors:** Huy Quang Duong, Shinya Kajiura, Tien Dinh Truong, Minh Tuan Ngo, Khai Xuan Nguyen, Hung Khanh Pham, Thang Ba Ta, Dung Tien Nguyen, Ryuji Hayashi

**Affiliations:** a Department of Gastroenterology, 103 Military Hospital, Vietnam Military Medical University, Hanoi, Vietnam; b Department of Clinical Oncology, Toyama University Hospital, Toyama, Japan; c Department of Pathology, 103 Military Hospital, Vietnam Military Medical University, Hanoi, Vietnam; d Center for Diagnostic Imaging, 103 Military Hospital, Vietnam Military Medical University, Hanoi, Vietnam; e Oncology Center, 103 Military Hospital, Vietnam Military Medical University, Hanoi, Vietnam; f Respiratory Center, 103 Military Hospital, Vietnam Military Medical University Hanoi, Vietnam

**Keywords:** Fatty liver disease, Multifocal fatty liver nodules, Nonalcoholic fatty liver disease

## Abstract

Multifocal fatty liver nodules can present a diagnostic challenge due to their resemblance to metastatic liver disease. This case report illustrates the complexity of such scenarios through the presentation of a middle-aged male patient. Despite the common nature of fatty liver disease, characterized by hepatocyte fat accumulation leading to diffuse and uniform liver lesions, rare instances exhibit heterogeneous appearances. The case underlines the potential confusion arising from imaging modalities when multiple small nodules disperse throughout the liver, mimicking multifocal tumors or metastases. The report emphasizes the critical role of comprehensive diagnostic procedures in preventing misdiagnosis and unwarranted interventions. Effective management hinges on multidisciplinary collaboration among specialists, ensuring accurate differentiation and appropriate treatment. This study serves as a reminder of the intricacies involved in interpreting multifocal fatty liver nodules that may masquerade as metastatic disease, highlighting the need for precision in clinical practice.

## Introduction

Fatty liver disease is a common clinical condition characterized by fat accumulation in hepatocytes. It usually presents as diffuse and uniform lesions throughout the liver. In rare cases, it may present with a heterogeneous appearance [1]. When it appears as multiple small nodules scattered throughout the liver, imaging modalities can lead to the confusion of the diagnosis with that of multifocal tumors or metastases. In this report, we present the case of a middle-aged male patient with multifocal fatty liver nodules to highlight the possibility of misinterpretation of this presentation based on imaging methods in daily practice.

## Case presentation

**Fig. 1:**
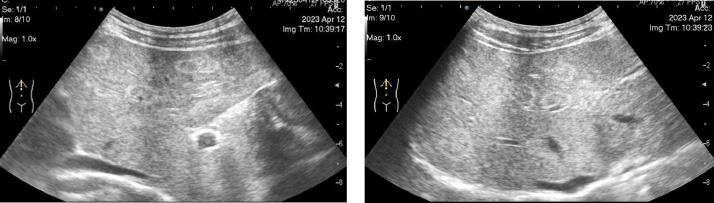
Liver ultrasound reveals multiple centimetric nodules scattered throughout the liver parenchyma.

A 50-year-old male patient presented with incidental multifocal liver nodules identified on abdominal ultrasound (US) during a regular health checkup. The round, homogenous nodules ranging from 0.9 to 1.5 cm in diameter, were distributed throughout the liver parenchyma and were isoechoic with a clear hyperechoic rim (Fig. 1). He had no remarkable medical history of liver disease, alcohol or drug consumption, or malignancies. The patient denied experiencing any symptoms associated with systemic diseases, such as weight loss, fatigue, or digestive dysfunction. Physical examination showed a body mass index within the normal range (21.2 kg/m^2^), with stable vital signs and no evidence of jaundice or abdominal masses. However, because of the concerning appearance of the identified liver nodules, the patient was admitted to the hospital for further investigation.

Laboratory findings revealed a mild elevation of serum cholesterol (6.98 mmol/L) and triglycerides (2.59 mmol/L), with reference ranges of <5.2 mmol/L and <2.25 mmol/L, respectively. He had normal test results for complete blood count and liver function (alanine transaminase, aspartate transaminase, prothrombin time, serum bilirubin, total protein, and albumin levels). Serological markers for Hepatitis B and C were negative. Levels of tumor markers, including alpha-fetoprotein, carcinoembryonic antigen, carbohydrate antigen 19-9, and cytokeratin-19 fragment were within the reference limits.

**Fig. 2:**
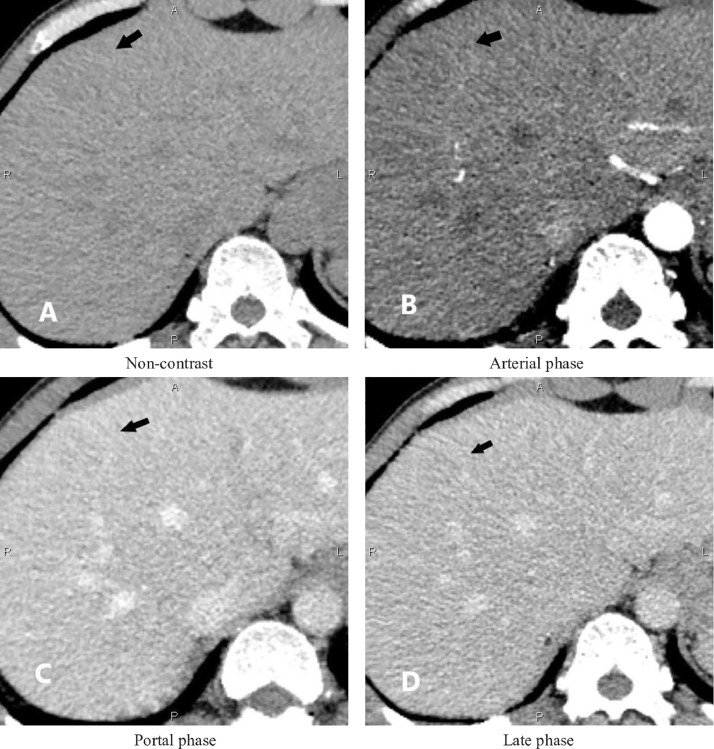
Contrast-enhanced CT reveals multiple hypoattenuating lesions (A) without enhancement in the arterial phase (B), the portal phase (C), and the late phase (D) (arrow).

**Fig. 3:**
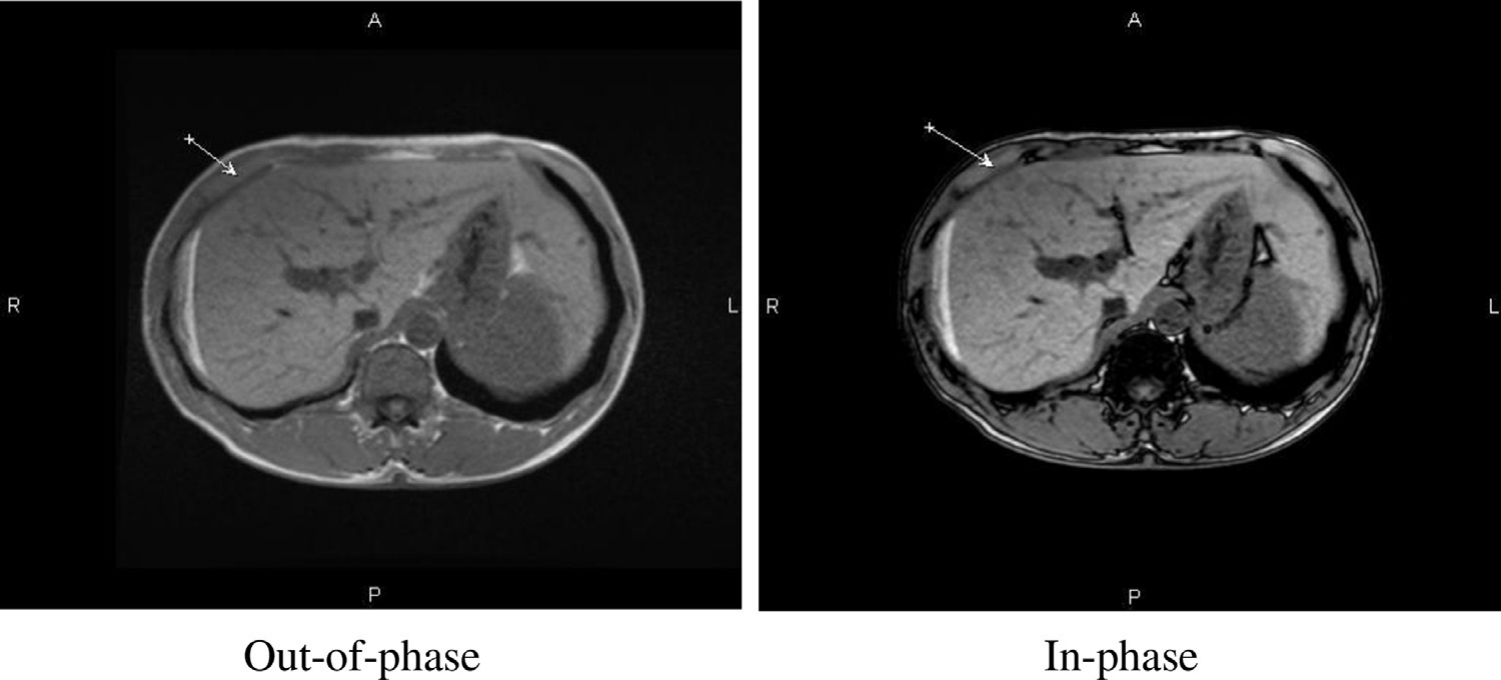
MRI shows hypointense hepatic foci (arrow) with decreased signal intensity on the out-of-phase image (B) compared to the in-phase image (A), indicating intralesional fatty substance.

Corresponding to the sonographic appearance of the liver, computed tomography (CT) revealed multiple hypoattenuating lesions without enhancement after the administration of a contrast agent (Fig. 2). This finding can be attributed to the malignant nature of the nodules. The patient subsequently underwent abdominal contrast-enhanced magnetic resonance imaging (MRI). The foci were hypointense on opposed-phase T1 and showed a decrease in signal intensity on out-of-phase images, which indicates a high possibility of intralesional fatty substances (Fig. 3). The 2 imaging modalities showed no evidence of mass effect on the liver surface, hepatic vein, or bile duct. No free intraperitoneal fluid or abdominal tumors were observed.

**Fig. 4:**
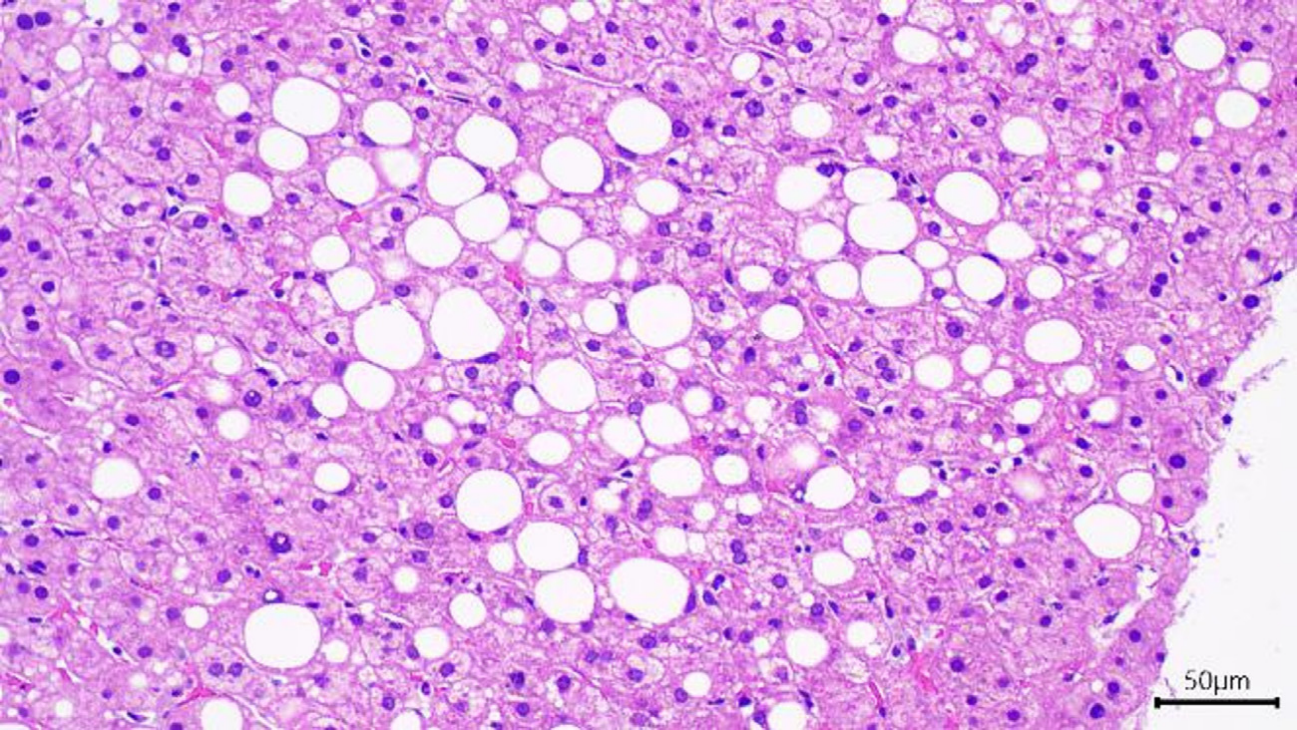
The US-guided liver biopsy sample shows that the liver architecture is maintained. There is an accumulation of fats in the cytoplasm of the hepatocytes. No primary or metastatic lesions were found (Hematoxylin & Eosin stain, 200x).

To give a definitive conclusion and rule out other possibilities, some invasive diagnostic methods were necessary. Colorectal and gastrointestinal endoscopies did not reveal any lesions. An ultrasound-guided liver biopsy was therefore performed. The biopsy specimens revealed that the liver was structurally unchanged. A prominent microscopic feature was intracytoplasmic fatty droplets in the liver cells, without inflammation or liver cell damage. The biopsy also eliminated the initial suspicion of primary liver tumors or metastases (Fig. 4).

Based on these findings, the lesions were confirmed as multifocal fatty liver nodules. The patient was referred to a hepatologist and advised to make lifestyle changes to improve his cholesterol and triglyceride levels. Periodic follow-up imaging was also recommended to monitor nodule progression.

## Discussion

Hepatic steatosis is characterized by excessive aggregation of fat in more than 5% of the hepatocytes [2]. Since it is closely related to alcohol consumption, obesity, and common metabolic disorders, it is becoming an increasingly serious health problem in many countries. Nonalcoholic fatty liver disease is the predominant type, with a prevalence of approximately 30% among adults worldwide [3]. Our patient had high cholesterol and triglyceride levels, which are among the most common risk factors for nonalcoholic fatty liver disease [4].

Clinically, identifying aggregation of fat in the liver is based on noninvasive imaging methods, the most frequently used of which is US, which is comparable to histology [5]. Fatty liver parenchyma typically shows diffuse hyperechogenicity and beam attenuation, resulting in poorly visualized intrahepatic vessels and bile ducts [6].

On CT scans, it appears hypodense on noncontrast-enhanced images [7]. In addition, MRI has been proven to be a powerful tool for evaluating steatosis. A drop in signal intensity on out-of-phase images compared with in-phase images confirms the presence of fatty liver [8].

In most cases, the diagnosis of hepatic steatosis is unequivocal because of the dominant diffuse pattern and disease prevalence. Less commonly, the fatty liver exhibits focal fat deposition or focal fatty sparing [9]. Multinodular fatty liver is an uncommon pattern with multiple small, well-demarcated lesions scattered throughout the liver parenchyma; several previous cases have been reported in the literature [10,11]. The underlying cause of multinodular fatty liver is not well understood, but has been linked to several factors, including autoimmune disorders, drug toxicity, and infections [12]. Multiple lesions can increase the risk of misdiagnosing multifocal liver tumors or metastases. In our case, the initial sonogram showed foci of centimetric nodules with a hyperechoic rim, which shared the US features, but not the characteristics, of some conditions, including hemangiomas, regenerative nodules, focal nodular hyperplasia, and metastatic tumors [13]. The definitive diagnosis, therefore, is challenging and requires a multidisciplinary approach, particularly in adults or patients with known malignancies. In terms of differentiating multiple fatty lesions from hepatic tumors, our patient presented with several suggestive features, including small nodules, hypointensity with minimal enhancement on contrast CT, and an absence of mass effect [1]. In addition, a significant signal drop on opposed-phase images compared with in-phase images on MRI is a clear indication of the presence of intracellular fat [14]. In this case, contrast-enhanced MRI using agents like gadoxetate sodium was not performed, although it might have been beneficial for making the diagnosis without biopsy.

Despite the negative findings of the laboratory investigations and imaging studies, suspicion of liver tumors and metastases remained, and a liver biopsy was recommended. Histopathological examination is the gold standard for a conclusive diagnosis. In this case, US-guided core needle biopsy was performed, which revealed intracytoplasmic deposition of fatty droplets and confirmed the absence of primary or metastatic cancer.

Treatment of this condition involves lifestyle modifications such as weight loss, exercise, and dietary changes to improve metabolic parameters and reduce the accumulation of fat in the liver. Regular follow-up imaging is also recommended to monitor the progression of liver nodules. In this case, a short-term repeat of the scan may have been useful for diagnosis without biopsy, and this approach should be considered in cases of biopsy refusal.

## Conclusion

Multifocal fatty liver nodules can be challenging to treat because they resemble metastatic liver disease. A thorough work-up is crucial to avoid misdiagnosis and unnecessary treatment. Multidisciplinary collaboration among specialists is necessary to diagnose and manage this condition accurately.

## Patient consent

We have obtained written informed consent from the patient for the publication of this case report. The patient consented to de-identified clinical information and images being used for the purposes of this report. The authors of the manuscript retain this informed consent and can provide it to the journal upon specific request.
